# Supplementary material for: Essential Oils of Alpinia nantoensis Retard Forskolin-Induced Melanogenesis via ERK1/2-Mediated Proteasomal Degradation of MITF
Source: Plants (Basel). 2020 Nov 28;9(12):1672. doi: 10.3390/plants9121672 (PMC7760488; doi:10.3390/plants9121672)
Supplement: Supplementary file 1 [file plants-09-01672-s001.pdf]

Supplementary Information

RESEARCH ARTICLE

Essential oils of *Alpinia nantoensis* retard forskolin-Induced melanogenesis *via* ERK1/2-mediated proteasomal degradation of MITF

K. J. Senthil Kumar [1,2], **M. Gokila Vani**[1], **Pei-Chen Wu**[1], **Hui-Ju Lee**[1], **Yen-Hsueh Tseng**[1], **Sheng-Yang Wang**[1,3,*]

[1] Department of Forestry, National Chung Hsing University, Taichung, Taiwan

[2] Center for General Education, National Chung Hsing University, Taichung, Taiwan

[3] Agricultural Biotechnology Research Institute, Academia Sinica, Taipei, Taiwan

Correspondence

Prof. Sheng-Yang Wang, Department of Forestry, National Chung Hsing University, 250, Kou Kuang Rd, Taichung 40227, Taiwan. Phone Number: +886-422850333; Fax Number: +886-422862960

E-mail address: taiwanfir@dragon.nchu.edu.tw (S-Y Wang)

Supplementary Table 1. List of antibodies used in this study

S. No	List of Antibodies	Host	Source
Primary antibodies			
1	ERK1/2	Rabbit	Cell Signaling Technology, Danvers, MA
2	Phos-ERK1/2	Rabbit	
3	AKT	Rabbit	
4	Phos-AKT	Mouse	
5	MITF	Rabbit	Abcam, Cambridge, UK
6	TRP-1	Mouse	Santa-Cruz Biotechnology, Dallas, TX
7	TRP-2/DCT	Mouse	
8	GAPDH	Mouse	
9	Ub	Rabbit	
10	Tyrosinase	Rabbit	GeneTex, Irvin, CA
11	CREB	Rabbit	
12	Phos-CREB	Rabbit	
Secondary antibodies			
1	Anti-Mouse IgG (HRP-linked)		Cell Signaling Technology, Danvers, MA
2	Anti-Rabbit IgG (HRP-linked)		
3	Anti-Mouse IgG (FITC-conjugated)		Alexa Fluor 488,<br>ThermoFisher Scientific, Waltham, MA
4	Anti-Rabbit IgG (FITC-conjugated)		

Supplementary Table 2.

qRT-PCR primer sequences		
**Gene**	**Forward**	**Reverse**
**TYR**	5'-TATTGAGCCTTACTTGGAAC-3'	5'-AAATAGGTCGAGTGAGGTAA-3'
**TRP-1**	5'-TGCAGGAGCCTTCTTTCTC-3'	5'-AAGACGCTGCACTGCTGGTCT-3'
**TRP2/DCT**	5'-GGATGACCGTGAGCAATGGCC-3'	5'-CGGTTGTGACCAATGGGTGCC-3'
**MITF**	5'-GTATGAACACGCACTCTCTCG-3'	5'-CTTCTGCGCTCATACTGCTC-3'
**GAPDH**	5'-TCAACGGCACAGTCAAGG-3'	5'-ACTCCACGACATACTCAGC-3'

Figure S1

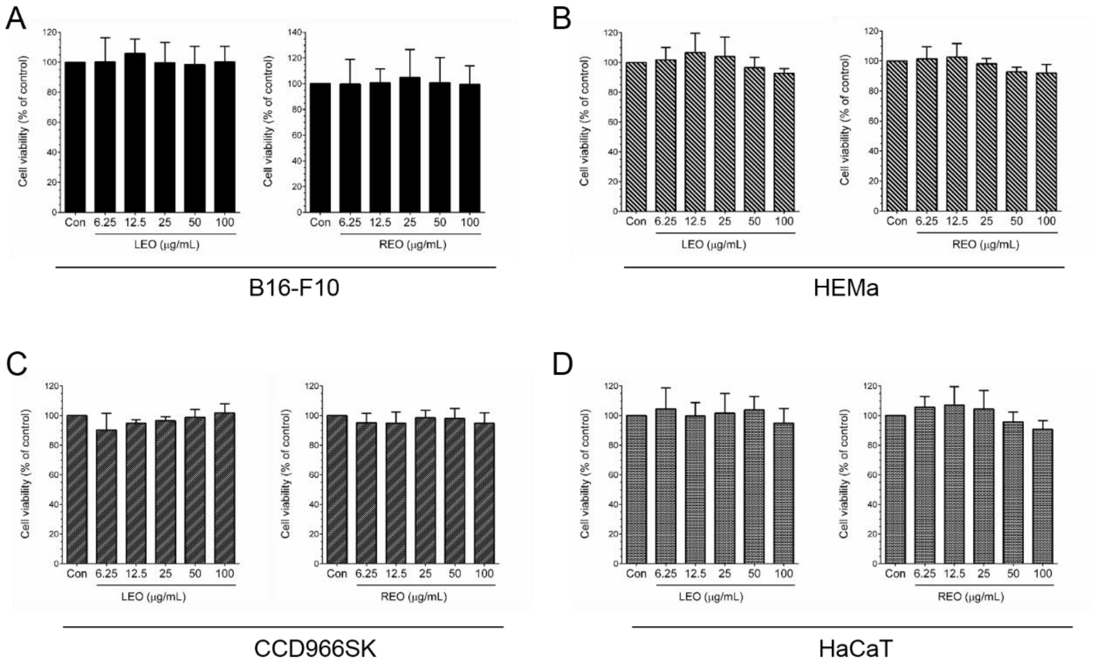

Figure S1. Cytotoxic effect of LEO and REO on dermal cell lines. Murine melanoma B16-F10, Human epidermal melanocytes-adult (HEMa), human skin fibroblasts CCD966SK and human skin keratinocytes were incubated with increasing concentrations of LEO or REO for 48 h. Cell viability was measured by MTT assay. The percentage of viable cells was compared with untreated control group. Results are expressed as mean ± SD of three independent experiments.

Figure S2

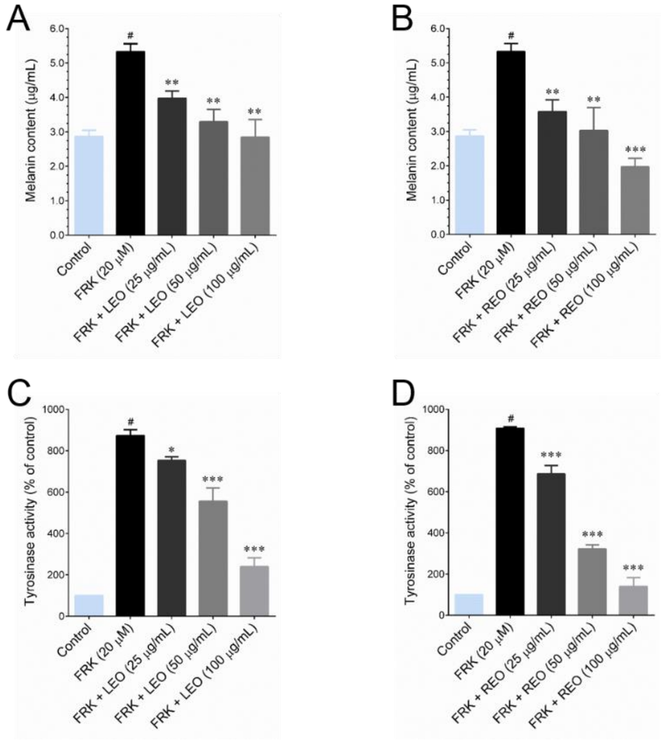

Figure S2. Inhibitory effect of LEO and REO on melanin content and tyrosinase enzyme activity in FRK-induced B16-F10 melanoma cells. Cells were treated with increasing concentrations of LEO and REO and stimulated with FRK for 48 h. Melanin content was assessed by with an absorbance at 405 nm. (A, B) Cellular melanin content was calculated by comparison with a melanin standard curve. (C, D) Effect of cellular tyrosinase activity was determined using whole cell lysates. After treatment with indicated concentrations of LEO and REO for 48 h. Cell lysates were used as enzyme source and L-DOPA as substrate. The effects on L-DOPA oxidation velocity was measured at 492 nm. The percentage of tyrosinase activity was compared with untreated control group. Data represent the mean ± SD of three experiments. Statistical significance was set at $^{\#}P < 0.05$ compared to control *vs.* FRK and *P < 0.05, **P < 0.01, ***P < 0.001 compared with FRK + sample treatment groups *vs*. control group.

Figure S3

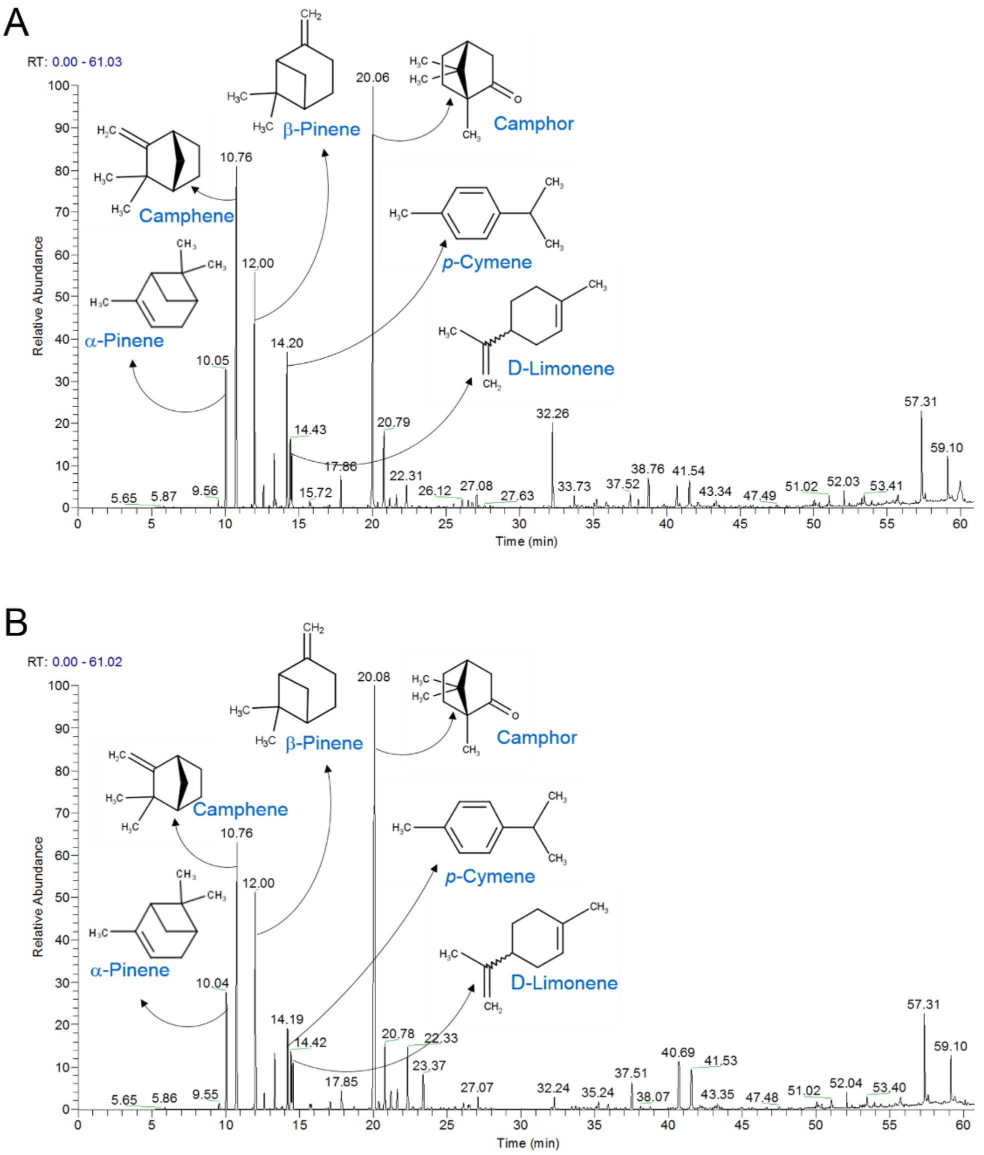

Figure S3. GC–MS analysis of LEO and REO. GC profiles and chemical structures of the major compounds in LEO (A) and REO (B) of *A. nantoensis*.

Figure S4

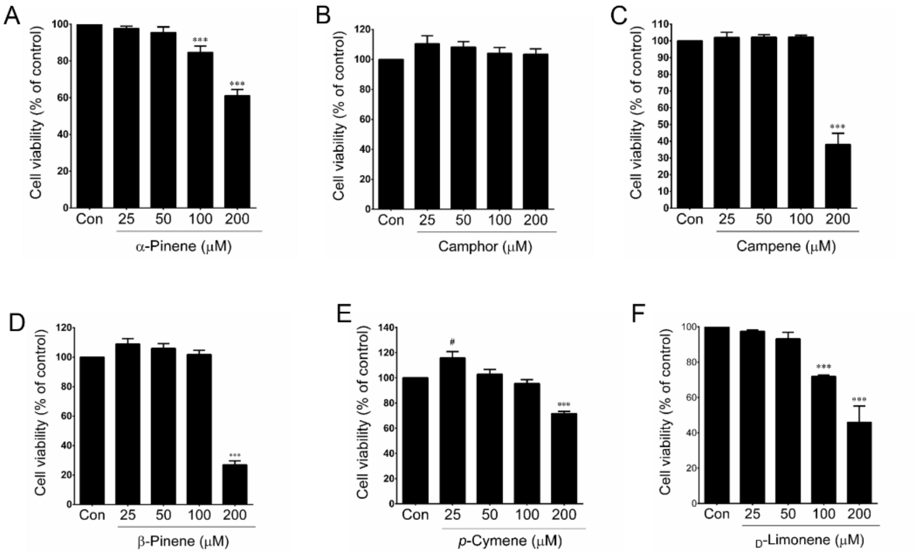

Figure S4. Cytotoxic effect of test compounds on B16-F10 cells. Murine melanoma B16-F10 cells were incubated with increasing concentrations of test compounds for 48 h. Cell viability was measured by MTT assay. The percentage of viable cells was compared with untreated control group. Results are expressed as mean ± SD of three independent experiments.
